# Population‐Level Trends in Gastric Cancer Mortality and Incidence After Expansion of Japanese National Health Insurance Coverage for *Helicobacter pylori* Eradication: A Descriptive Time‐Trend Study

**DOI:** 10.1111/hel.70162

**Published:** 2026-09-08

**Authors:** Masahiro Asaka, Tokuma Tanuma, Akiko Kowada, Shouji Matsushima, Kozo Akino

**Affiliations:** ^1^ Caress Memorial Hospital Sapporo Japan; ^2^ Department of Occupational Health Kitasato University Graduate School of Medical Sciences Sagamihara Kanagawa Japan; ^3^ Department of Cardiovascular Medicine Kyushu University Graduate School of Medicine Fukuoka Japan; ^4^ Member of the House of Councilors Tokyo Japan

**Keywords:** chronic gastritis, eradication therapy, gastric cancer, *Helicobacter pylori*, Japan, national health insurance

## Abstract

**Background:**

In February 2013, Japanese national health insurance coverage for *Helicobacter pylori* eradication was expanded to patients with *H. pylori* ‐positive, endoscopically diagnosed chronic gastritis. This policy broadened access to eradication treatment, but its relationship with subsequent population‐level gastric cancer trends requires cautious interpretation.

**Aim:**

To describe national trends in gastric cancer mortality and incidence before and after the 2013 insurance expansion, with attention to age‐specific patterns, endoscopic screening, treatment uptake, and alternative explanations.

**Methods:**

We examined publicly available national mortality data from the Ministry of Health, Labour and Welfare and cancer incidence data from the National Cancer Center. Calendar‐year trends in annual gastric cancer deaths, incident cases, and age‐specific deaths were evaluated descriptively. The 2013 policy change was treated as a major contextual event rather than as a causal intervention with an internally estimated counterfactual.

**Results:**

Annual gastric cancer deaths declined from 48,427 in 2013 to 37,394 in 2024, a 25.2% reduction in crude annual deaths. Incidence also decreased after a later peak, with available national incidence data showing fewer new cases by 2021. Age‐specific analyses showed pronounced decreases in deaths among people younger than 80 years, whereas deaths among those aged 80 years or older continued to increase.

**Conclusion:**

After the 2013 expansion of insurance coverage for *H. pylori* eradication, gastric cancer mortality and incidence in Japan declined at the population level. These ecological trends are compatible with a contribution from increased eradication therapy and endoscopy‐linked detection, but causality cannot be established from aggregate data alone. Age‐standardized, claims‐linked, stage‐specific, and treatment‐linked analyses are needed to quantify the independent policy effect.

## 1 Introduction

Gastric cancer remains a major public health problem despite long‐term declines in incidence and mortality in many countries. *Helicobacter pylori* is the principal infectious cause of non‐cardia gastric cancer and has been classified as a Group 1 carcinogen by the International Agency for Research on Cancer (IARC) [1, 2]. International attention has therefore shifted from treatment of *H. pylori* ‐related peptic ulcer disease alone toward population‐level strategies for gastric cancer prevention [2, 3].

Japan provides a distinctive setting because of its high historical burden of gastric cancer, high use of upper gastrointestinal endoscopy, and universal health insurance system. Before 2013, national insurance coverage for *H. pylori* eradication was limited mainly to peptic ulcer disease and selected conditions, including gastric mucosa‐associated lymphoid tissue lymphoma, idiopathic thrombocytopenic purpura, and after endoscopic resection of early gastric cancer [4, 5]. In February 2013, coverage was expanded to include *H. pylori* ‐positive chronic gastritis diagnosed by endoscopy. This change did not constitute an organized population screening program. Rather, it created an endoscopy‐linked clinical pathway in which many patients with chronic gastritis could receive reimbursed eradication therapy after *H. pylori* testing [6].

This article uses the term “endoscopy‐linked insurance expansion for *H. pylori* eradication” rather than “Search and Treat,” because the latter may imply active population screening. This distinction is important because opportunistic endoscopy, organized endoscopic screening, treatment uptake, background *H. pylori* prevalence, birth‐cohort replacement, dietary change, smoking patterns, treatment advances, and registry practices may all influence population‐level gastric cancer trends [6, 7, 8, 9].

Previous studies have examined the effect of the 2013 insurance expansion on eradication treatment uptake and *H. pylori* prevalence using claims, prescription, and survey data [6, 7]. The aim of this study was different: to provide a decadal descriptive overview of national gastric cancer mortality and incidence trends after the 2013 insurance expansion and to discuss how these trends should and should not be interpreted. We specifically examined whether changes differed by age group, because Japan's rapidly aging population can obscure trends in population risk when crude counts are used [10, 11].

## 2 Methods

### 2.1 Design and Setting

We conducted a descriptive, population‐based national time‐trend study of gastric cancer mortality and incidence in Japan. The principal contextual event was the February 2013 expansion of national health insurance coverage for eradication therapy in patients with *H. pylori* ‐positive chronic gastritis confirmed by endoscopy.

### 2.2 Data Sources

Mortality data were obtained from publicly available vital statistics of the Ministry of Health, Labour and Welfare. Incidence data were obtained from publicly available cancer statistics compiled by the National Cancer Center. National census and population data were used for age‐specific interpretation where applicable. The study used aggregate, anonymized public data and did not involve individual‐level patient records.

### 2.3 Outcomes

The outcomes were annual gastric cancer deaths, annual gastric cancer incidence, and age‐specific gastric cancer deaths. Crude annual counts are presented because they directly describe the national disease burden faced by the health system. However, crude counts are not equivalent to population risk. Age‐specific analyses were therefore used to clarify how the observed national burden differed across generations. Age‐standardized mortality and incidence rates are the preferred metrics for estimating changes in population risk and for international comparison [11, 12].

### 2.4 Statistical Approach

The submitted dataset supported descriptive trend analysis rather than causal inference. We therefore interpreted the 2013 policy change as a major policy milestone and did not estimate a formal counterfactual policy effect. No chi‐squared tests comparing pre‐ and post‐policy national counts were used because such tests are not informative in complete national registry data and do not address causality. Joinpoint or segmented regression can be useful for identifying changes in trend, but results should be reported with annual percent change estimates and confidence intervals and should be tested in sensitivity analyses using alternative intervention years and latency periods. Because stage distribution, treatment, survival, and individual eradication histories were not linked in the present aggregate dataset, mechanisms of mortality reduction were considered hypotheses rather than demonstrated pathways.

### 2.5 Ethics

This study used anonymized, publicly available national statistics. Institutional review board approval and informed consent were not required under applicable Japanese ethical guidance for analyses of public aggregate data.

## 3 Results

### 3.1 National Mortality Trends

**FIGURE 1:**
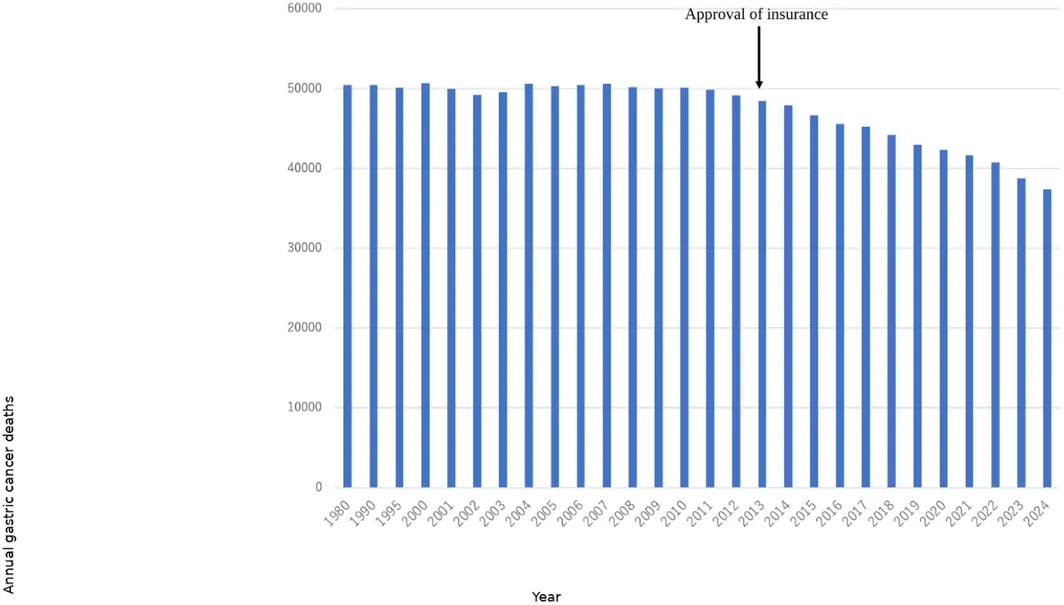
Annual gastric cancer deaths in Japan, 1980–2024. The *y*‐axis shows annual crude gastric cancer deaths, and the *x*‐axis shows calendar year. Annual deaths remained close to 50,000 for many years and declined after 2013. The arrow indicates the expansion of national health insurance coverage for *Helicobacter pylori* eradication in *H. pylori* ‐positive chronic gastritis.

**FIGURE 2:**
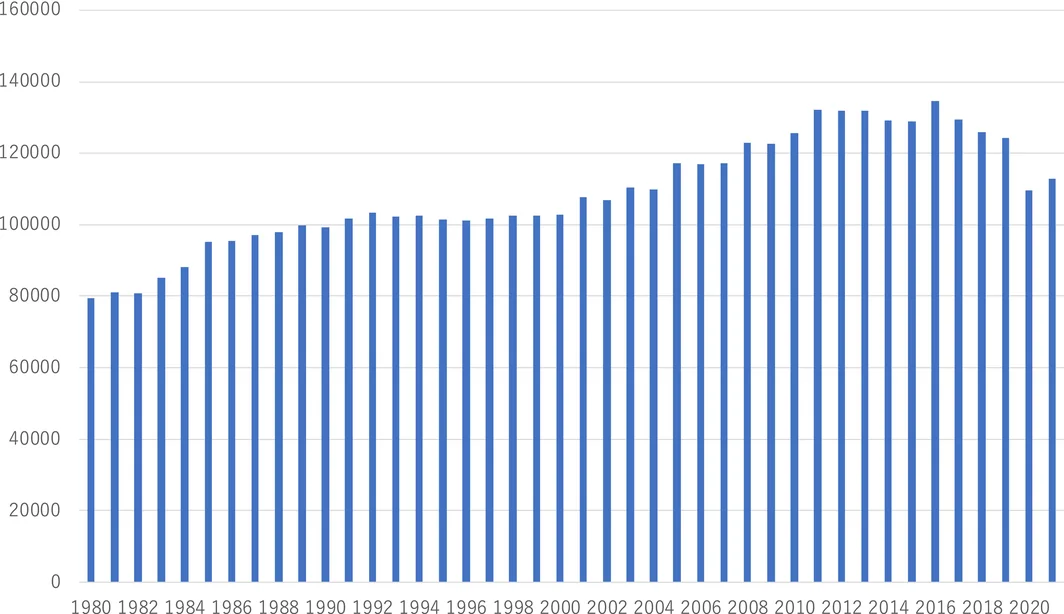
Annual gastric cancer incidence in Japan, 1980–2021. The *y*‐axis shows annual incident gastric cancer cases, and the *x*‐axis shows calendar year. National annual incident cases rose through the early 2010s and decreased in later available years. These crude counts should be interpreted alongside age‐standardized rates and registry context.

The *y*‐axis shows annual crude gastric cancer deaths, and the *x*‐axis shows calendar year. Annual deaths remained close to 50,000 for many years before declining after 2013. The arrow indicates the expansion of national health insurance coverage for *H. pylori* eradication in patients with *H. pylori* ‐positive chronic gastritis (Figure 1). This decline occurred in the context of a preceding long‐term decline in gastric cancer risk, concurrent demographic change, and changes in detection and treatment. National incidence trends. The *y*‐axis shows annual incident gastric cancer cases, and the *x*‐axis shows calendar year. National annual incident cases rose through the early 2010s and decreased in the most recent available years. These crude counts should be interpreted alongside age‐standardized rates and registry context (Figure 2). The timing is compatible with several mechanisms, including eradication‐related prevention in susceptible groups, changes in detection and diagnostic pathways, aging of high‐risk birth cohorts, secular decline in *H. pylori* prevalence, dietary change, and registry‐related factors. The aggregate data cannot distinguish these explanations.

### 3.2 Age‐Specific Mortality Patterns

**FIGURE 3:**
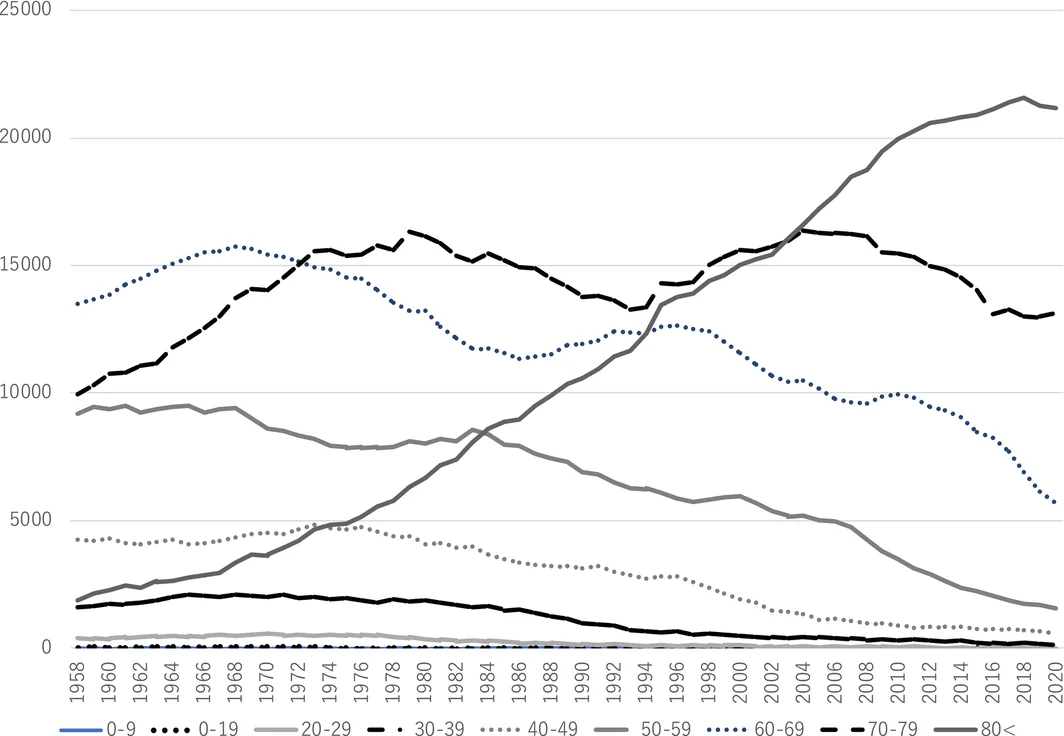
Age‐specific annual gastric cancer deaths in Japan, 1958–2020. The *y*‐axis shows annual gastric cancer deaths, and the *x*‐axis shows calendar year. Deaths declined in most age groups younger than 80 years, whereas deaths among people aged 80 years or older increased, reflecting demographic and cohort effects.

The *y*‐axis shows annual gastric cancer deaths, and the *x*‐axis shows calendar year. Deaths declined in most age groups younger than 80 years, whereas deaths among people aged 80 years or older increased, reflecting demographic and cohort effects (Figure 3). Deaths declined sharply among people younger than 80 years, including middle‐aged and early elderly groups. In contrast, deaths among people aged 80 years or older continued to rise over much of the study period. This divergence indicates that the national burden reflects both changing disease risk and the rapid growth of Japan's oldest age groups. It also supports the need to evaluate age‐standardized rates and age‐specific outcomes rather than relying solely on crude national counts [10, 11].

### 3.3 Context of Eradication Uptake and Endoscopy

Earlier reports indicate that eradication therapy increased markedly after the 2013 insurance expansion, and clinical practice required endoscopic diagnosis of chronic gastritis before insurance‐covered treatment [6, 7]. It is therefore plausible that both increased eradication therapy and increased endoscopic detection contributed to subsequent changes in gastric cancer burden. However, the present dataset did not include individual treatment histories, first‐line or second‐line prescription records linked to outcomes, eradication success or failure, endoscopy counts linked to cancer outcomes, stage at diagnosis, or survival. The relative contribution of each pathway cannot therefore be quantified in this study.

## 4 Discussion

This descriptive national analysis shows that gastric cancer mortality and incidence declined in Japan after the 2013 expansion of national insurance coverage for *H. pylori* eradication in chronic gastritis. The main value of the present study is not to prove a policy effect, but to place the post‐2013 national trends in clinical and demographic context. The central finding is that the decline in crude national deaths after 2013 was accompanied by a striking divergence by age group: deaths decreased in people younger than 80 years, whereas deaths among those aged 80 years or older continued to rise [10, 11].

These findings are consistent with, but do not prove, a contribution from the 2013 insurance expansion. Several mechanisms could plausibly have acted together. First, expanded access to eradication therapy may have reduced future cancer development among infected individuals whose gastric mucosa had not reached an advanced irreversible stage [3, 4]. Second, the requirement for endoscopic diagnosis before reimbursed treatment may have increased detection of prevalent early cancers or high‐risk mucosal changes. Third, improved endoscopic treatment and oncologic care may have contributed to better survival. Fourth, long‐term birth‐cohort replacement, falling background *H. pylori* prevalence, reduced salt intake, changes in smoking, and other lifestyle factors likely contributed to the broader decline [7, 13]. Because these factors changed simultaneously, a single national ecological time series cannot separate them.

Endoscopic screening is an especially important co‐intervention. Endoscopic gastric cancer screening was introduced into the national screening framework in the mid‐2010s, overlapping with the post‐2013 period. Experience from South Korea has shown that the expansion of endoscopic screening can change stage distribution and mortality trends independent of primary prevention. Therefore, the temporal decline in Japanese gastric cancer mortality should be interpreted as the result of a combined prevention and detection environment rather than eradication therapy alone. Where available, future analyses should stratify radiographic screening, endoscopic screening, overall screening participation, and age‐specific participation rates.

Treatment uptake after 2013 is central to interpretation. Claims and pharmaceutical‐sales analyses reported a substantial increase in eradication therapy after insurance coverage was expanded to chronic gastritis, and survey‐based data suggest declining *H. pylori* prevalence in Japan [6, 7]. However, the present dataset did not contain annual first‐line prescriptions, second‐line prescriptions, confirmation testing, eradication success rates, or retreatment rates linked to cancer outcomes. Such information is needed to determine whether the insurance‐based pathway was implemented uniformly by age group and region and whether reductions in gastric cancer burden track the intensity and success of eradication therapy.

Antibiotic resistance and broader consequences of mass eradication also require monitoring. Clarithromycin resistance is a recognized cause of eradication failure, and widespread antibiotic exposure may influence subsequent treatment success, the microbiome, and antimicrobial stewardship concerns [14, 15, 16]. The introduction of more potent acid suppression, including vonoprazan‐based regimens, may improve eradication rates in some settings, but it does not remove the need for surveillance of antimicrobial resistance, repeated treatment, and adverse outcomes [16]. The present ecological analysis cannot evaluate microbiome changes, multidrug‐resistant infections, pneumonia mortality, or extra‐gastric diseases after eradication therapy. These issues have therefore been added as limitations and as priorities for post‐policy surveillance.

The age‐specific findings have practical implications. The continued rise in deaths among people aged 80 years or older may reflect the large size of older birth cohorts, high historical *H. pylori* exposure, advanced atrophic gastritis or intestinal metaplasia at the time eradication becomes available, lower screening participation, higher comorbidity burden, competing mortality, and decisions to forego invasive treatment after cancer diagnosis. This pattern supports earlier risk assessment and treatment before severe mucosal damage has occurred, but it does not justify claims that testing young adults would eliminate gastric cancer entirely. Such strategies require feasibility, cost‐effectiveness, antibiotic‐resistance, adverse‐event, and adherence evaluations [8, 9, 12].

The updated international context also requires a measured interpretation. The 2025 IARC Working Group report and the 2026 *New England Journal of Medicine* summary support population‐based *H. pylori* screen‐and‐treat strategies as a promising approach for gastric cancer prevention, while emphasizing implementation quality, antibiotic stewardship, local disease burden, monitoring, and equity [12, 17]. Japan's experience is informative because it demonstrates how a universal insurance system can broaden access to eradication therapy, but it should be presented as one model among several rather than as definitive proof of a universally replicable policy effect.

This study has several limitations. First, it is ecological and descriptive; individual exposure to eradication therapy was not measured. Second, the analysis did not include a control group or an internally validated counterfactual, so it cannot estimate the number of deaths prevented by the policy. Third, crude counts are influenced by population aging and should be supplemented by age‐standardized rates. Fourth, formal Joinpoint analysis with annual percent changes and confidence intervals was not performed because the necessary age‐standardized rates files were not available for a fully reproducible analysis. Fifth, stage distribution, treatment patterns, and survival data were unavailable, limiting mechanistic interpretation. Sixth, screening participation, endoscopy utilization, antibiotic resistance, extra‐gastric outcomes, and registry definitions may have changed over time. These limitations are substantial and should temper the conclusions.

Despite these limitations, the observed post‐2013 trends are clinically important. They show that the national burden of gastric cancer in Japan has declined below the long‐standing plateau of approximately 50,000 annual deaths and that this decline was concentrated among people younger than 80 years. The findings support continued monitoring of *H. pylori* eradication uptake, antibiotic resistance, gastric cancer incidence, stage distribution, screening participation, and age‐standardized mortality.

## 5 Conclusion

Japan experienced a substantial decline in gastric cancer deaths and a later decline in incidence after the 2013 expansion of national insurance coverage for *H. pylori* eradication in chronic gastritis. These population‐level trends are compatible with a beneficial contribution from broadened eradication therapy and endoscopy‐linked detection, but the ecological design cannot establish causality or quantify deaths prevented. The Japanese experience should be interpreted as an important real‐world case study that warrants further age‐standardized, claims‐linked, screening‐linked, resistance‐monitoring, and stage‐specific evaluation.

## Author Contributions

**Masahiro Asaka:** conceptualization, methodology, investigation, writing – original draft, and project administration. **Tokuma Tanuma:** data curation and resources. **Akiko Kowada:** formal analysis and methodology. **Shouji Matsushima:** validation and investigation. **Kozo Akino:** conceptualization and writing – review and editing. All authors read and approved the final manuscript.

## Funding

The authors have nothing to report.

## Conflicts of Interest

The authors declare no conflicts of interest.

## Data Availability

All data used in this analysis were derived from publicly available national statistics from the Ministry of Health, Labour and Welfare and the National Cancer Center of Japan.
